# Factors contributing to the use of traditional herbal medicine among hospitalised high-risk pregnant women in Namibia

**DOI:** 10.4102/hsag.v31i0.3508

**Published:** 2026-07-24

**Authors:** Foibe T. Teofilus, Alice Lifalaza, Daniel O. Ashipala

**Affiliations:** 1 Department of General Nursing Science, Faculty of Health Sciences and Veterinary Medicine, University of Namibia, Windhoek, Namibia

**Keywords:** traditional herbs, maternal health, pregnancy, antenatal care, cultural beliefs, Namibia

## Abstract

**Background:**

Traditional herbs are widely used during pregnancy, especially in developing countries, influenced by cultural beliefs and accessibility. Pregnant women often use these remedies to relieve discomfort. Despite ongoing debate about their safety and efficacy, little is known about factors influencing their use, highlighting the need for further research and clearer clinical guidance to ensure maternal and foetal safety.

**Aim:**

The purpose of this study was to explore and describe the factors contributing to the use of traditional herbs among pregnant women at Rundu Intermediate Hospital, Kavango East Region, Namibia.

**Setting:**

The study was conducted in the maternity wards of an intermediate hospital in Namibia.

**Methods:**

A qualitative, explorative, descriptive design was employed. Data were collected through semi-structured interviews guided by open-ended questions. Fifteen pregnant women were purposively sampled based on inclusion criteria.

**Results:**

Four main themes emerged from the data analysis: reasons for using traditional herbs during pregnancy, perceived benefits of traditional herbs, motivating factors influencing the use of traditional herbs and strategies to address the use of traditional herbs.

**Conclusion:**

The study concluded that pregnant women’s widespread use of traditional herbal remedies is motivated by cultural beliefs, perceived health benefits and accessibility. Accordingly, it would appear that pregnant women may have insufficient awareness of potential risks.

**Contribution:**

Findings from this study can be used to implement culturally responsive health education on the risks and consequences of the use of herbal remedies, as well as to strengthen prenatal promotion programmes to ensure safe and integrated maternal care outcomes.

## Introduction

Herbal treatments and traditional medicine have long been central to healthcare systems. While modern health systems are mainly based on scientific methods, many now recognise and integrate traditional practices as complementary care (Mortada 2024). Through research and regulation, traditional medicine is increasingly accepted within formal health structures. Herbal therapy has its historical roots in ancient Chinese, Ayurvedic and Indigenous healing traditions and is widely considered a natural and affordable substitute for traditional medications (Saggar et al. 2022). Traditional treatments are still frequently utilised today, particularly in maternity care in some low- and middle-income countries such as Peru and parts of sub-Saharan Africa, despite advances in modern medicine and the evidence-based approach of formal healthcare systems (Cabada-Aguirre et al. 2023). According to the World Health Organization (WHO) (Poli et al. 2025), up to 80% of people in underdeveloped countries such as Ethiopia, India and parts of sub-Saharan Africa rely on traditional medicine as their primary source of medical care, including pregnancy-related services. In Ethiopia, herbal treatments are frequently used to induce labour, support foetal health and relieve pregnancy-related discomforts such as nausea, vomiting and exhaustion (Belayneh, Yoseph & Ahmed 2022). However, toxicity, negative drug interactions and possible injury to both mother and child are among the serious hazards associated with the unregulated nature of herbal medicine and the absence of defined dosages (Hassen et al. 2022).

The extensive use of herbal medicine during pregnancy in many parts of the world is being highlighted by an expanding body of research. An Iranian study found that over 40% of Asian expecting mothers reported using herbal medication, often because of family traditions and beliefs about the effectiveness of natural remedies (Vardanjani et al. 2023). Traditional Chinese Medicine, which incorporates herbal decoctions, remains a major part of maternity care in China, despite concerns about the potential teratogenic effects of some herbs (Wang & Chen 2025). According to studies, between 10% and 55% of pregnant women in Europe use herbal remedies, depending on cultural background and access to healthcare. These remedies are often self-administered at home rather than taken within hospital settings or under professional supervision (Josendal & Bergmo 2024). Notably, despite ongoing debate regarding their efficacy and safety, herbal remedies such as ginger and raspberry leaf continue to be widely used during pregnancy in the UK, highlighting the need for further research and clearer clinical guidance (Sarecka-Hujar & Szulc-Musioł 2022). In many parts of Africa, pregnant women often turn to traditional medicine because of various contributing factors such as limited access to formal healthcare, cultural beliefs, affordability and trust in indigenous practices (Thipanyane et al. 2022).

In addition, based on cultural beliefs, trust in traditional healers and the perceived safety of natural remedies, herbal treatments are frequently used during pregnancy. In many communities, traditional healers are primarily sought for curative or spiritual interventions, while traditional birth attendants (TBAs) focus on providing care and support for pregnant women before, during and after childbirth. This distinction is important for understanding the various factors influencing traditional medicine use in maternal healthcare (Thipanyane et al. 2022). Research conducted in Ghana and Nigeria indicates that many women utilise herbal remedies because they are dissatisfied with the maternity care system, are on a limited budget, or are influenced by elders or TBAs (Osseo-Asare 2023). In Uganda, research indicates that over 80% of home births involve the use of herbal remedies, some of which contain oxytocic compounds that can induce labour but may also lead to complications such as uterine rupture and foetal distress (Perlman 2025). South African studies have also raised concerns over the use of herbal preparations intended to ease childbirth but which have been linked to adverse pregnancy outcomes, including uterine hyperstimulation and neonatal complications (Dlamini, Darong & Nkwanyana 2023). Namibia, like many other countries, continues to experience the use of traditional herbal treatments among pregnant women, particularly in the Kavango East Region, despite the availability of modern obstetric care (Ministry of Health and Social Services [MoHSS] 2023). Some expectant mothers consult traditional healers and TBAs, either as a primary source of care or in conjunction with biomedical services (Mudonhi & Nunu 2022; Toit & Pretorius 2018). This practice is often influenced by ingrained cultural beliefs, perceived trust and familiarity with traditional practitioners, and limited access to formal healthcare facilities, especially in rural and semi-urban areas (Mudonhi & Nunu 2022).

While certain herbal remedies may provide symptomatic relief, their use remains concerning owing to the lack of scientific validation, unregulated dosages and the potential toxicity of some herbs (Okaiyeto & Oguntibeju 2021). In Namibia, a degree in complementary therapies – including herbal use – is not offered at University. Hence, this study aimed to explore the factors contributing to the use of traditional herbs among pregnant women.

## Research methods and design

This study employed a qualitative, exploratory and descriptive research design to explore the factors influencing the use of traditional herbs among pregnant women in the maternity ward at Rundu Intermediate Hospital. This approach was deemed suitable for gaining an in-depth understanding of lived experiences and complex phenomena within a real-world context (Brink, Van der Walt & Van Rensburg 2018). An exploratory design helps uncover patterns in under-researched topics (Hay et al. 2020), while a descriptive design provides a detailed account of the phenomenon (Ampomah et al. 2021). This approach helped to generate valuable insights to inform maternal healthcare practices and policy interventions.

### Setting

The study was conducted at Rundu Intermediate Hospital, a regional referral hospital located in the Kavango East Region of Namibia. This hospital provides healthcare services for both urban and rural populations and offers a broad range of services including general medicine, surgery, paediatrics and maternal and child health. Specifically, data were collected from the antenatal section of the maternity ward, where pregnant women are admitted for monitoring and observations, management of high-risk pregnancies and care prior to labour. This section manages conditions such as pregnancy-induced hypertension, gestational diabetes, treatment for other diagnoses during pregnancy and complications requiring intrapartum monitoring. With over 200 pregnant women accessing intrapartum and postpartum care at the hospital each month, and a significant number admitted before delivery, this setting was ideal for exploring the factors influencing the use of traditional herbs during pregnancy.

### Population and sample

The target population was comprised of pregnant women admitted to the antenatal section of the maternity ward at Rundu Intermediate Hospital in the Kavango East Region of Namibia during the study period. Rundu Intermediate Hospital was selected because it serves as the regional referral hospital for the Kavango East, Kavango West and Zambezi regions and manages a substantial number of deliveries and high-risk pregnancies.

Women admitted to the antenatal ward were screened for eligibility with the assistance of ward staff. Only pregnant women who reported having used traditional herbs during the current pregnancy, were clinically stable, not in active labour, not experiencing severe pain, and able to provide informed consent were considered eligible for participation. Women who were in active labour, medically unstable, experiencing severe discomfort or unable to participate in an interview were excluded. A total of 18 pregnant women were screened for eligibility during the study period. Out of the 18 women screened, three women were medically unstable and therefore excluded from participation. Fifteen women met the inclusion criteria, were considered eligible for inclusion, and consented to participate in the study.

Purposive sampling was employed to recruit information-rich participants who had first-hand experience with traditional herb use during pregnancy. Particular attention was given to women nearing delivery and those referred from peripheral clinics for ongoing antenatal management, as these women were more likely to report traditional medicine use. Conducting interviews in the antenatal section of the maternity ward enabled the inclusion of this key group while ensuring that only women in a stable condition were approached.

The final sample consisted of 15 pregnant women who met the inclusion criteria and agreed to participate in the study. Sample size was guided by the principle of data saturation, defined as the point at which no new information emerged from subsequent interviews. Saturation was achieved by the tenth interview; however, interviews continued until participant 15 to ensure richness of the data and confirm thematic redundancy.

As the study utilised purposive sampling within a qualitative design, the intention was not to achieve statistical representativeness but rather to obtain in-depth information from participants with direct experience of traditional herb use during pregnancy.

### Data collection

The study employed semi-structured, one-on-one interviews guided by an interview schedule that met the study objectives. It is advised that semi-structured interviews be conducted, as this allows more flexibility, while still allowing consistency between interviews (Demirci 2024). To facilitate free expression among participants, interviews were conducted in Rukwangali. With the permission of participants, all interviews were audio-recorded. Data were collected through individual interviews conducted in a private and comfortable environment, a hospital boardroom, over a 2-month period, from August 2025 to September 2025.

Each interview lasted approximately 30 min to 45 min and, to ensure that no valuable information was lost, interviews were audio-recorded with the participants’ permission. In addition, the researcher took field notes to capture key observations and contextual insights during the interviews. The interview guide served as a framework for guiding the interview process, ensuring that key aspects of the research question were covered.

A pilot test was initially conducted with four participants from the same sampling unit as the actual study setting to determine the relevance and clarity of the interview questions. These participants met the same criteria as the target population. No changes were made to the interview guide following the pilot study, and the data obtained from the pilot interviews were not included in the main study.

The primary researcher (FT) posed the following core open-ended questions, with follow-up probes used to explore participants’ responses in greater depth:

- *Can you tell me more about why you opted to use traditional herbs during pregnancy?*
- *What do you think are the benefits of using traditional herbs during pregnancy?*
- *What do you think motivates pregnant women to use traditional herbs during pregnancy?*
- *What do you think can be done to address the use of traditional herbs during admission to the maternity ward at Rundu Intermediate hospital?*

### Data analysis

Raw data from the audio recordings were transcribed verbatim before being analysed using Braun et al.’s (2019) six steps of data analysis. Firstly, the primary researcher (FT) familiarised himself with the data by rereading interview transcripts, listening to the recordings multiple times and taking notes. Secondly, initial codes were generated to describe the content and assign labels to features of the data that were relevant to the research question. The primary researcher (FT) personally transcribed and coded the data, as he was familiar with the study objectives and research questions. Using a coded Microsoft document, in vivo codes were generated through a meticulous process of reading each word and line, and highlighting relevant portions of the data using distinct colours. In addition, concise labels were applied to enhance clarity when referring to specific codes in all the transcripts. Subsequently, the researcher systematically searched for, sorted and grouped related codes into potential themes on the basis of participants’ thoughts, views, understandings and interpretations of the factors contributing to the use of herbs by pregnant women. Thirdly, themes were searched for in the data from all the interview transcripts by reviewing the coded data to identify areas of similarities and overlap. Fourthly, potential themes were then reviewed by checking them against the organised extracts of the data to determine whether the themes worked in relation to the dataset. Fifthly, themes were defined and named by summarising each theme in a few sentences to produce a coherent and comprehensive account of the data. The themes were further reviewed for relevance and clarity and tested for referential adequacy by returning to the raw data. Thereafter, the themes were refined, and succinct thematic descriptions were written as unique stories. An independent coder, holding a Doctorate in Nursing Education and specialising in qualitative research, conducted an autonomous thematic analysis of the data. The findings were then cross-referenced with the primary researcher (FT) analysis to ensure validation. Units of significance were classified and subcategorised based on participants’ viewpoints, ultimately leading to the identification of themes.

Participants were uniquely coded from P1 to P16, and pseudonyms were created for face-to-face interview participants, followed by a number. Finally, a report was produced by writing a clear, coherent and compelling description of the data based on the analysis. The researcher and an independent coder identified and agreed on the main themes.

### Trustworthiness

Trustworthiness refers to the degree of confidence in the data, procedures and interpretations employed to guarantee the quality of the study (Ahmed 2024). The researcher applied the following criteria proposed by Lincoln and Guba (1985): credibility, dependability, transferability and confirmability. Ensuring trustworthiness is crucial in establishing the credibility and reliability of qualitative findings, given their subjective nature (Peddle 2022). These measures were implemented throughout the research process as follows: Credibility was established by ensuring a rigorous data collection process and maintaining prolonged engagement with the participants for 16 weeks. The researcher built trust by thoroughly explaining the purpose of the study and ensuring participants felt comfortable sharing their experiences. Member checking was conducted, where participants were given an opportunity to review their interview transcripts to verify the accuracy of their responses. Peer debriefing was also utilised to gain alternative perspectives and interpretations, thus enhancing the credibility of the findings. Transferability was ensured by providing a detailed description of the research context, study setting and participants’ characteristics. This thick description allows readers to assess whether the findings are applicable to similar contexts or populations.

Verbatim quotes from participants were included to provide deeper insight into the use of traditional herbs among pregnant women. Dependability was addressed by maintaining a clear and detailed audit trail of all research activities. The methodology, data collection tools and analysis processes of the study were thoroughly documented, ensuring that the research could be replicated by others. An independent coder was involved in the thematic analysis to cross-check the consistency of the coding process and interpretations. To ensure confirmability, the researcher actively practised reflexivity by maintaining a journal to record personal biases, assumptions and reflections throughout the study. This minimised the potential influence of the researcher’s subjective views on the findings. In addition, all data, including audio recordings, transcripts and codes, were reviewed by a second researcher to verify the accuracy and neutrality of the interpretations. Truth value was emphasised by ensuring that participants’ voices were represented accurately and respectfully. By including diverse participants, the study captured a range of perspectives and experiences, contributing to a holistic understanding of the use of traditional herbs among pregnant women. By rigorously applying these measures, the study findings were deemed trustworthy and thus provide valuable insights into the use of traditional herbs among pregnant women.

### Ethical considerations

Ethical approval was obtained from the University of Namibia’s School of Nursing and Public Health. The ethical clearance number is SoNREC 207/2025. Ethical clearance was also received from the Ethics Committee and the MoHSS’ (Ref No. 22/4/2/3). Participants were provided with detailed information about the study purpose and procedures, as well as their rights as participants, including the right to withdraw at any time. Written informed consent was obtained from all participants before the interviews commenced. Confidentiality was maintained by assigning pseudonyms to participants and securely storing all data. Audio recordings and transcripts were kept in password-protected files, accessible only to the research team. At the end of the study, the data were stored securely for a period of 5 years and will then be destroyed in accordance with institutional policy. The researcher adhered to the following fundamental ethical principles: autonomy, beneficence, non-maleficence, justice and respect for human dignity.

## Results

### Demographic characteristics of study participants

A total of 15 pregnant women participated in the study. Participants’ ages ranged from 20 to 34 years. The majority of participants were married (*n* = 10), while five were single. Regarding educational attainment, seven participants had completed primary education, five had completed secondary education and three had attained tertiary education. Most participants resided in rural areas (*n* = 11), while four resided in urban areas. Regarding occupation, five participants were employed, five were unemployed, three were self-employed and two were students. In terms of parity, four participants had one child, seven had between two and three children, and four had between four and five children. All participants identified as Christian. All participants were in their third trimester of pregnancy and reported using traditional herbs during their current pregnancy (see Table 1).

**TABLE 1:** Demographic characteristics of study participants (*N* = 15).

Participant code	Age (years)	Marital status	Occupation†	Parity	Religion	Education level	Residence
P1	23	Married	Unemployed	2	Christian	Primary	Rural
P2	28	Single	Self-employed	3	Christian	Secondary	Rural
P3	21	Single	Student	1	Christian	Secondary	Urban
P4	32	Married	Unemployed	4	Christian	Primary	Rural
P5	25	Married	Employed	2	Christian	Secondary	Urban
P6	30	Single	Unemployed	3	Christian	Primary	Rural
P7	27	Married	Employed	2	Christian	Secondary	Rural
P8	29	Married	Self-employed	4	Christian	Primary	Rural
P9	20	Single	Unemployed	1	Christian	Primary	Rural
P10	26	Married	Employed	2	Christian	Secondary	Urban
P11	24	Single	Student	1	Christian	Tertiary	Urban
P12	22	Married	Unemployed	1	Christian	Primary	Rural
P13	33	Married	Self-employed	5	Christian	Primary	Rural
P14	34	Married	Employed	4	Christian	Tertiary	Rural
P15	28	Married	Employed	3	Christian	Tertiary	Rural

† , Employed outside home.

### Themes and sub-themes that emerged from the data analysis

During the data analysis, four main themes emerged from the participants’ responses, supported by sub-themes derived from their experiences and views. These themes were developed to address the research objectives and the open-ended interview questions (see Table 2).

**TABLE 2:** Themes and sub-themes.

Themes	Sub-themes
1. Socio-cultural factors contributing to the use of traditional herbal medicine among pregnant women	1.1 Cultural practice influence from older family members 1.2 Beliefs in herbal cleansing as a fast effect on labour process 1.3 Beliefs in strengthening overall health of the foetus
2. Accessibility and affordability of traditional herbs	2.1 Accessibility of traditional herbs 2.2 Affordability of traditional herbs
3. Perceived strategies to address the use of traditional herbs among pregnant women	3.1 Health education on risks of traditional herbs 3.2 Collaboration between healthcare workers and traditional healers 3.3 Strengthening antenatal health promotion programmes

### Theme 1: Socio-cultural factors contributing to the use of traditional herbal medicine among pregnant women

This theme describes the reasons why pregnant mothers chose to use traditional herbs despite having access to modern healthcare. The findings revealed that socio-cultural beliefs, family influence and the desire for faster childbirth were the main reasons for herb use.

#### Sub-theme 1.1: Cultural practice influence from older family members

Participants reported that using traditional herbs during pregnancy is deeply rooted in their cultural heritage.

The majority of participants were influenced by practices passed on from older family members and considered them to be a normal, respected part of their identity and traditions:

‘In our community, using herbs during pregnancy is something our mothers and grandmothers have always done. It is believed to help protect the baby from evil spirits and make the birth easier. If you do not use them, some people think your baby will suffer or something bad might happen.’ (P3, 21 Years, student)

‘My mother-in-law insisted that I drink the herbs she gave me. She said all her daughters used them and had no complications. I didn’t want to upset her, so I followed what she said, even though the nurse at the clinic warned me.’ (P2, 28 Years, self-employed)

#### Sub-theme 1.2: Beliefs in herbal cleansing as a fast effect on labour process

Many participants believed that traditional herbs shortened labour and made delivery easier. They perceived the herbs as preparing the womb and facilitating a smooth birth, reducing pain and the risk of complications. This practice was often influenced by advice from elders and peers who had used herbs successfully in previous pregnancies. This is supported by the following quotes from two participants:

‘The herbs help clean the womb by removing dirt and things that can cause infection or delay the baby’s movement. My grandmother told me that after taking them, the womb becomes ready for the baby to come out.’ (P4, 32 Years, unemployed)

‘I started taking the herbs in my eighth month because my neighbour said they make the baby come out faster. I don’t want to suffer for many hours when giving birth, so I just followed her advice.’ (P5, 25 Years, employed)

#### Sub-theme 1.3: Belief in strengthening the overall health of the foetus

Participants believed that traditional herbs promote the unborn baby’s strength and overall health. Many felt that herbs support proper foetal growth, enhance activity in the womb, and protect the baby from weakness or illness. This belief was reinforced by advice from elders and previous positive experiences in the community. Some participants expressed the following:

‘They say when you drink certain herbs, the baby becomes strong and active. I noticed my baby moved more after I started using them, and I believed it was working.’ (P1, 23 Years, unemployed)

‘My mother told me that herbs make the baby’s bones strong and prevent weakness after birth. That is why I continued using them even after coming to the hospital.’ (P12, 22 Years, unemployed)

### Theme 2: Accessibility and affordability of traditional herbs

Participants reported that accessibility, low cost and advice from elders or traditional healers strongly influenced their use of herbs. Limited trust in hospital medications also led many to prefer traditional remedies. These perspectives show that convenience, cultural endorsement and perceived reliability make herbal use a common choice during pregnancy.

#### Sub-theme 2.1: Accessibility of traditional herbs

The availability of traditional herbs makes herbs a practical choice for many women, especially those with limited financial resources or who have difficulty accessing formal healthcare. Participants verbalised this as follows:

‘You can get herbs from any old woman in the village, and they are very cheap.’ (P13, 33 Years, self-employed)

‘You just go to the forest, or someone gives them to you for free.’ (P3, 21 Years, student)

‘Herbs are also easily available in the village from traditional healers, you don’t need to pay transport cost to go to the hospital.’ (P14, 34 Years, employed)

#### Sub-theme 2.2: Affordability of traditional herbs

Participants reported that traditional herbs are inexpensive compared to hospital medicines. In addition, mothers stated that they can be obtained locally from families, elders or the environment without much cost:

‘The herbs are easy to find from elder women and traditional healers and do not cost much.’ (P15, 28 Years, employed)

‘Hospital medicine is expensive, and sometimes they tell us to buy from pharmacies.’ (P13, 33 Years, self-employed)

‘I believe the natural things are better than chemicals, you get them from elder women for free in the village.’ (P7, 27 Years, employed)

### Theme 3: Perceived strategies to address the use of traditional herbs among pregnant women

Participants suggested improving health education, strengthening antenatal programmes and promoting collaboration between healthcare workers and traditional healers to ensure safer maternal practices and informed choices regarding herbal use.

#### Sub-theme 3.1: Health education on the risks of traditional herbs

Participants emphasised the need for healthcare workers to educate pregnant women about the potential risks of traditional herbs. They suggested that early and clear information during antenatal visits could help women make safer choices, prevent complications and reduce reliance on unverified herbal remedies during pregnancy:

‘Nurses should talk to us more during antenatal visits. Many women use herbs because they don’t know the dangers. If we are told early, we can stop using them.’ (P10, 26 Years, employed)

‘I think health education should be done in all villages so that even old women know the side effects. Sometimes we use herbs because no one explains the risks.’ (P9, 20 Years, unemployed)

#### Sub-theme 3.2: Collaboration between healthcare workers and traditional healers

Participants suggested that collaboration between nurses and traditional healers could improve maternal health outcomes. By combining medical knowledge with cultural practices, women could receive safe guidance on herbal use. This partnership would help reduce risks, build trust and ensure that traditional remedies are used responsibly during pregnancy:

‘Hospitals should work with traditional healers, so they can teach which herbs are safe. This will help both sides to understand each other.’ (P6, 30 Years, unemployed)

‘If traditional healers are trained by nurses, they can help guide us correctly instead of giving dangerous herbs.’ (P11, 24 Years, student)

#### Sub-theme 3.3: Strengthening antenatal health promotion programmes

Participants emphasised the importance of enhancing antenatal health promotion through community outreach and educational talks. They suggested that regular programmes could raise awareness about the risks of traditional herbs, encourage safer pregnancy practices and empower women to make informed choices while fostering engagement between healthcare providers and the community. Two participants made the following recommendations:

‘Health workers should come to the villages often to educate women. Most of us do not get enough information unless we are admitted.’ (P12, 22 Years, unemployed)

‘Antenatal classes should include topics about herbs and cultural practices so that women can make better choices.’ (P8, 29 Years, self-employed)

## Discussion

### Participant characteristics

This study explored factors influencing the use of traditional herbs during pregnancy among women admitted to the antenatal section of the maternity ward at Rundu Intermediate Hospital. The participants were between 20 and 34 years of age, with most being married, having fewer than three live births, and with varied occupational backgrounds. All participants reported using traditional herbs during their current pregnancy. Their experiences highlighted the continued importance of traditional medicine in maternal healthcare and provided insight into the socio-cultural, economic and healthcare-related factors that influence use.

### Themes

Three main themes emerged from the study: socio-cultural factors influencing traditional herb use, accessibility and affordability of traditional herbs, and strategies to address traditional herb use during pregnancy.

### Socio-cultural factors influencing traditional herb use during pregnancy

The study revealed that pregnant women use traditional herbs despite access to modern healthcare, primarily as a result of cultural beliefs, family influence and the desire for easier childbirth. These findings underscore the persistence of traditional practices in maternal care, reflecting deep-rooted cultural identities that influence health decisions. Participants’ accounts highlight how herbs are not merely alternatives but integral to cultural narratives of protection and continuity. This theme demonstrated how traditional medicine sustains community bonds and addresses perceived inadequacies in formal healthcare systems.

Findings were consistent with those of Chekero (2024), which found that Zimbabwean migrant women in South Africa blended traditional medicine with religious approaches to navigate maternal and infant health threats, emphasising the role of herbs in spiritual and physical safeguarding. Similarly, Haikera (2021) examined cultural practices in Namibia’s Kavango regions, noting that herbs are woven into pregnancy rituals to ensure familial and communal well-being, aligning with the observations of the current study regarding the intergenerational transmission of knowledge.

Socio-cultural beliefs emerged as a key driver, with refusal to use herbs often regarded as a breach of social norms. This social enforcement concurs with the findings of Mudonhi and Nunu (2022), who found that familial expectations compelled pregnant women to adhere to traditional remedies, often prioritising harmony over individual health risks. Thipanyane et al. (2022) similarly reported that traditional practices are upheld through community expectations, creating a dynamic in which advice from elders may take precedence over professional healthcare guidance.

The desire for a smooth and quick delivery further amplified herb use, as participants recounted the perceived efficacy of herbs in facilitating labour based on anecdotal successes. Ramkumar (2025) highlighted similar preferences among East African women for traditional birth attendants who use herbs to expedite deliveries. Likewise, Sawyer (2024) reported that herbs are frequently promoted for their perceived practical benefits despite potential risks.

Participants also perceived traditional herbs as natural, safe and beneficial for maternal and foetal health, particularly for cleansing, strengthening and pain reduction. Cabada-Aguirre et al. (2023) similarly documented the use of traditional medicines in reproductive health for cleansing and protection. Drebka et al. (2025) further support these findings through reports of perceived benefits of herbal products for gynaecological conditions. Participants believed that herbs strengthened the unborn baby and enhanced foetal development, findings that align with Balarastaghi et al. (2022), who reported similar perceptions among pregnant women.

The belief that herbs reduced labour pain and shortened labour and birth was also evident. Zangeneh and Hantoushzadeh (2023) reported that traditional herbs are commonly believed to facilitate uterine contractions and labour progression. Similar findings were documented by Ssenku et al. (2022) in Eastern Uganda, where medicinal plants are frequently used to support labour and childbirth. These findings reveal a gap between perceived and evidence-based benefits. Sarecka-Hujar and Szulc-Musioł (2022) cautioned that the safety of herbal medicines during pregnancy remains uncertain and that some herbs may have adverse maternal or foetal effects. This highlights the need for educational interventions that respectfully address cultural beliefs while promoting evidence-based maternal healthcare.

### Accessibility and affordability of traditional herbs

Accessibility, affordability, elder influence and mistrust of hospital medications motivated the use of traditional herbs, positioning them as practical alternatives for pregnant women. These factors highlight systemic barriers in healthcare access and trust, where economic and social realities often favour traditional practices over modern medicine. The findings are consistent with Belayneh et al. (2022), who reported that affordability and local availability are major factors influencing herbal medicine use among pregnant women in Ethiopia. Similarly, Mudonhi and Nunu (2022) found that the widespread availability of herbs in low-resource settings contributed significantly to continued use.

Elderly women and traditional healers emerged as trusted authorities whose advice was highly valued by participants. Ampomah et al. (2021) reported similar findings in Ghana, where traditional healers played an influential role in maternal healthcare decision-making. Rutledge et al. (2024) further supported the importance of traditional birth attendants in maternal care within low- and middle-income countries.

Limited trust in hospital medications also contributed to herb use. Participants often viewed pharmaceuticals as less desirable than natural remedies. Brown et al. (2024) observed that medical mistrust can lead individuals to favour natural treatments over conventional medicines, while Webb Hooper et al. (2022) linked healthcare mistrust to increased reliance on traditional alternatives among underserved populations. Collectively, these findings underscore broader healthcare equity challenges. As highlighted by Amzat and Razum (2021), inequalities in access to trusted and culturally acceptable healthcare may reinforce reliance on traditional medicine practices.

### Strategies to address traditional herb use during pregnancy

Participants suggested enhancing health education, fostering collaboration between healthcare workers and traditional healers, and strengthening antenatal programmes as key strategies to reduce the risks associated with traditional herb use during pregnancy. These recommendations reflect the need for integrated approaches that balance cultural practices with evidence-based care while promoting informed decision-making.

Choudhury et al. (2023) emphasised the importance of pharmacovigilance for herbal medicines, supporting participants’ recommendations for increased education regarding the potential risks associated with herbal use during pregnancy. Similarly, Sarecka-Hujar and Szulc-Musioł (2022) highlight ongoing uncertainties regarding herb safety, reinforcing the importance of awareness programmes during antenatal care.

Collaboration between healthcare workers and traditional healers emerged as another important recommendation. Ampomah et al. (2021) reported that integrating orthodox and traditional healthcare practices can improve maternal health outcomes. Likewise, Rutledge et al. (2024) advocate for the inclusion of traditional birth attendants within healthcare systems to promote safer maternal care.

Participants also recommended strengthening antenatal programmes through outreach and community engagement. These recommendations align with Namibia’s MoHSS (2023) maternal health initiatives and with Hannon et al. (2022), who emphasised the value of community-based health promotion programmes. Such approaches are consistent with (WHO 2013) recommendations on integrating traditional medicine into healthcare systems while ensuring patient safety and quality care.

### Study limitations and areas for further research

This study explored and described traditional herb use during pregnancy within a specific context, limiting the generalisation of findings to other regions or populations. The qualitative design relied on a small sample, potentially missing diverse perspectives. In addition, time constraints during data collection, caused by participants’ schedules, may have influenced the depth of responses. No effort was made to determine the herb(s) used during pregnancy, which should be determined in future studies. Future research could employ quantitative research to validate the prevalence and outcomes of traditional herb use during pregnancy. In addition, exploratory studies could be conducted to gather insights into patient and provider perspectives across broader healthcare settings to develop comprehensive strategies for integrating traditional and modern medicine.

### Recommendations

The following recommendations are derived from the study findings:

The MoHSS should strengthen health education during antenatal visits, ensuring that healthcare providers inform pregnant women about the potential risks of using traditional herbs. This approach may help reduce traditional herb use driven by cultural beliefs and empower pregnant women to make informed decisions. The MoHSS should also promote partnerships between nurses and traditional healers to integrate cultural knowledge with medical expertise, ensuring safe birth and reducing complications.

Community outreach programmes led by the MoHSS should include educational talks on the effects of traditional herbs during antenatal care. Such initiatives can empower pregnant women to make informed choices while addressing issues of accessibility, affordability and culturally supported practices. In addition, training institutions should incorporate cultural competence modules into nursing and medical education to build trust, address mistrust in hospital medications and improve communication with diverse patient populations. This will prepare healthcare professionals to engage respectfully with patients’ cultural practices, including addressing the use of traditional herbs.

## Conclusion

Participants perceived traditional herbs as playing a significant role in pregnancy care, often viewing them as natural remedies that address cultural, social and health needs. However, use of herbs may pose risks when not aligned with evidence-based practices. The purpose of this study was to explore and describe the use of traditional herbs during pregnancy. The findings revealed that pregnant women used these herbs as a result of cultural beliefs, family pressure and the desire for easier childbirth, perceiving benefits such as womb cleansing, foetal strengthening and pain reduction. Motivating factors included accessibility, affordability, elder influence and mistrust of hospital medications. Suggested strategies emphasised health education, collaboration between healthcare workers and traditional healers, and strengthened antenatal programmes to promote safer practices and informed decision-making.
